# Diagnostic value of the combined detection of serum tumor necrosis factor-α and fecal calprotectin in early sepsis-related encephalopathy

**DOI:** 10.3389/fmed.2025.1598624

**Published:** 2025-06-02

**Authors:** Chenghong OuYang, ChunLi Yang, Qi Wu, Fang Liu, Zhenhuan Chen

**Affiliations:** ^1^Department of Critical Care Medicine, Jiangxi Provincial People's Hospital, The First Affiliated Hospital of Nanchang Medical College, Nanchang, China; ^2^Department of Cardiology, Jiangxi Provincial People's Hospital, The First Affiliated Hospital of Nanchang Medical College, Nanchang, China

**Keywords:** serum tumor necrosis factor-α, fecal calprotectin, early sepsis-related encephalopathy, diagnostic value, pathogenesis of SAE

## Abstract

**Objective:**

To investigate the value of tumor necrosis factor-α (TNF-α) and fecal calprotectin in the early diagnosis and prognosis of sepsis-associated encephalopathy (SAE).

**Methods:**

We recruited 150 patients with sepsis, admitted from January 2020 to January 2022. Of these, 80 patients had SAE and 70 patients did not. The levels of serum TNF-**α** and fecal calprotectin of patients while in the intensive care unit were measured and correlated with the acute physiology and chronic health evaluation scoring system II (APACHE II) score, the sequential organ failure assessment (SOFA) score and 28-day mortality. We examined the value of TNF-α and fecal calprotectin in the diagnosis and prognosis of SAE.

**Results:**

The APACHE II and SOFA scores and 28-day mortality of the SAE group were significantly higher than those of the sepsis group, which indicated that the condition of the SAE group was more critical. In the SAE group, TNF-α and fecal calprotectin levels were positively correlated with the APACHE II and SOFA scores (*P* < 0.05), which may be related to disease severity. Assessing TNF-α level alongside fecal calprotectin level is highly valuable for the diagnosis of SAE and determining poor prognoses in SAE patients.

**Conclusions:**

TNF-α and fecal calprotectin may be involved in the pathogenesis of SAE. Both have high specificity and sensitivity for early SAE diagnosis. Moreover, they have good predictive value and can serve as prognostic indicators.

## 1 Introduction

Sepsis is a series of uncontrolled inflammatory reactions caused by infection, which leads to the dysfunction of multiple organs (1). Sepsis-associated encephalopathy (SAE) is one of the most common complications of sepsis; however, the specific underlying mechanism is currently unclear. The clinical manifestation of SAE is diverse and includes altered consciousness, psychosis, and delirium. Studies have shown that SAE is an independent risk factor for death in patients with sepsis (2).

Currently, there are no unified standards for the clinical diagnosis of SAE, and treatment options are limited. Therefore, in clinical practice, early screening, early diagnosis and prognosis evaluation of SAE patients are essential to reduce the incidence of and mortality due to SAE. Zhong et al. investigated the protective role of n-acetylcysteine in SAE using a rat model and monitored brain changes using magnetic resonance (MR) molecular imaging. They were the first to achieve non-invasive “dynamic visual monitoring” of physiological and pathological changes related to SAE using MR molecular imaging, providing a more sensitive imaging approach for early SAE diagnosis, identification and prognosis (3). Giga et al. administered a systemic injection of lipopolysaccharide (LPS) to mice for 24 h to examine the role of translocator protein 18 kDa (TSPO) in neuroinflammation and found that TSPO plays a critical role in the SAE mouse model. This suggested that monitoring TSPO activity and the progress of endotoxemia and its sequelae in animal models would deepen our understanding of the molecular mechanism underlying SAE (4). Another study conducted by Li et al. (5) showed that serum Tau protein, adrenocorticotropic hormone and cortisol have high clinical diagnostic value for SAE in burn patients with sepsis.

Tumor necrosis factor-α (TNF-α) has been shown to increase significantly in patients with SAE (6). In a sepsis model of Sprague Dawley rats induced via a caecal ligation and puncture procedure, it was demonstrated that the inflammatory response, including TNF-α, involves sepsis-induced changes in behavioral stereotypy (7), which suggests that TNF-α plays a vital role in the early prediction of SAE.

Calprotectin is a 36 kDa member of the S100 protein family. It is derived predominantly from neutrophils and has a direct antimicrobial effect on, and plays a role in, the innate immune response (8). Calprotectin has been implicated in inflammatory bowel disease (9) and rheumatoid arthritis (10). It is also known to contribute to vascular calcification in chronic kidney disease and is thus a potential therapeutic target (11). To date, no study has examined whether TNF-α level combined with calprotectin level can predict early SAE. Therefore, we retrospectively selected 150 patients with sepsis admitted from January 2020 to January 2022, comprising 80 patients with SAE and 70 patients without, to examine the diagnostic value of serum TNF-α level combined with fecal calprotectin level for early SAE.

## 2 Materials and methods

### 2.1 Patient characteristics

We implemented a retrospective research method and selected 150 sepsis patients who were admitted to the intensive care unit (ICU) of our hospital from January 2020 to January 2022 and met the Sepsis 3.0 diagnostic criteria (12), That is ① there must be one or more infections, including but not limited to pneumonia, peritoneal inflammation, urinary tract infection, skin and soft tissue infection, toxic shock, sepsis, etc. ② There must be two or more of the following symptoms: heart rate ≥90 beats per minute, respiratory rate ≥20 beats per minute, systolic blood pressure ≤ 100 mmHg. Diastolic blood pressure ≤ 60 mmHg, body temperature ≤ 36°C or ≥38°C, white blood cell count ≤ 4 ^*^ 10^9^/L or ≥12 ^*^ 10^9^/L. ③ There must be one or more organ dysfunction, including but not limited to heart failure, lung failure and liver failure, renal insufficiency, neurological insufficiency, etc. ④ There must be one or more metabolic disorders in the body. The study was reviewed and approved by our hospital ethics committee [Batch number: Kekuai 2024 (42)], and the participants or their authorized family members provided informed consent.

#### 2.1.1 Inclusion criteria

Patients were included if they met the Sepsis 3.0 diagnostic criteria (12), their SAE diagnosis conformed to the ICU Confusion Assessment Method (13) and they had no central nervous system infection.

#### 2.1.2 Exclusion criteria

Patients were excluded if they had undergone cardiopulmonary and cerebral resuscitation, or if they had had cerebrovascular accidents, intracranial organic lesions, other metabolic encephalopathies, inflammatory bowel disease, rheumatic immune system diseases or a history of malignant tumors.

#### 2.1.3 Collection of data

We collected the demographic and clinical data of the two groups of patients, which included age, sex, acute physiology and chronic health evaluation scoring system II (APACHE II) score, sequential organ failure assessment (SOFA) score, 28-day mortality and serum TNF-α and fecal calprotectin levels at admission.

#### 2.1.4 Main reagents and instruments

The TNF-α enzyme-linked immunosorbent assay (ELISA) kit was purchased from Qingdao Riskell Biotechnology of China. The Beckman Dxi800 fully automatic chemiluminescent immunoanalyzer was purchased from Beckman (US). The calprotectin detection kit was purchased from Weizheng Company. The portable immunoanalyzer WIZ-A101 was purchased from Xiamen Baysen Medical Technology of China.

### 2.2 Research methods

#### 2.2.1 Detection of TNF-α

We collected 5 mL of peripheral venous blood in a standard test tube from all patients when they were transferred to the ICU. The blood allowed to coagulate naturally at room temperature for 30 min. Then samples were then centrifuged at 1,000 × g for 10 min and separated. The serum was stored at 2°C−8°C and tested on the machine of the Beckman Dxi800 fully automatic chemiluminescent immunoanalyzer within 4 h. The TNF-α reagent using the ELISA and the operation was performed according the instructions.

#### 2.2.2 Detection of fecal calprotectin

We collected the feces (or enema) of all patients at admission send the samples to the department for testing. All operations are performed in accordance with the operating instructions.

### 2.3 Statistical methods

The SPSS 27.0 software was used for all statistical analyses. Data are expressed as means ± standard deviations (SDs), and *t*-tests were used for pairwise comparisons. Count data are expressed as frequency (%) and were analyzed using the chi-square test. Pearson correlation was used to analyse the correlation between serum TNF-α level, fecal calprotectin level, APACHE II score and SOFA score. The receiver operating characteristic curve (ROC) was used to evaluate the diagnostic value of TNF-α and fecal calprotectin levels for SAE. *P* < 0.05 was considered statistically significant.

## 3 Results

### 3.1 Group comparisons

**Figure 1:**
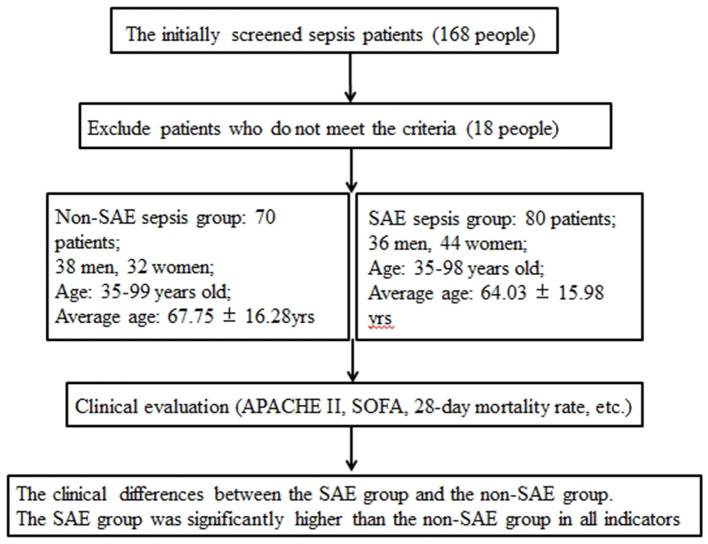
Flow chart showing inclusion process and outcomes of two groups.

Flow chart showing inclusion process and outcomes in Figure 1. There were 70 patients in the non-SAE sepsis group, comprising 38 men and 32 women aged 35–99 years (mean age 67.76 ± 16.28 years). In the SAE group, there were 80 patients with SAE, comprising 36 men and 44 women aged 35–98 years (mean age 68.66 ± 13.26 years). There was no significant difference in age or sex ratio between the two groups (*P* > 0.05). The APACHE II score (15.28 ± 7.08 vs. 18.18 ± 7.18), SOFA score (8.21 ± 5.94 vs. 10.35 ± 7.20), and 28-day mortality (0.20 vs. 0.49) rate of the SAE group were significantly higher than those of the non-SAE sepsis group (Table 1).

**Table 1:** Comparison of general information of the two groups of patients.

**Group**	**Male/Female**	**Age**	**APACHE II**	**SOFA**	**28-day mortality**
Sepsis	38/42	67.76 ± 16.28	15.28 ± 7.08	8.21 ± 5.94	0.20
SAE	36/44	68.66 ± 13.26	18.18 ± 7.18	10.35 ± 7.20	0.49
*t*/χ^2^	7.623	0.325	7.075	4.774	3.608
*P*	0.997	0.746	0.001	0.004	< 0.001

### 3.2 Group comparison of serum TNF-α and fecal calprotectin levels

The TNF-α (11.42 ± 9.39 vs. 20.17 ± 14.85) and fecal calprotectin levels (23.87 ± 17.96 vs. 53.99 ± 29.49) of the SAE group were significantly higher than those of the non-SAE sepsis group (Table 2).

**Table 2:** Comparison of serum TNF-α and fecal calprotectin levels between the two groups of patient.

**Group**	**TNF-α (pg/mL)**	**Fecal calprotectin (μg/g)**
Sepsis	11.42 ± 9.39	23.87 ± 17.96
SAE	20.17 ± 14.85	53.99 ± 29.49
*t*	5.105	9.508
*P*	0.002	0.001

### 3.3 Correlation between serum TNF-α and fecal calprotectin levels

**Figure 2:**
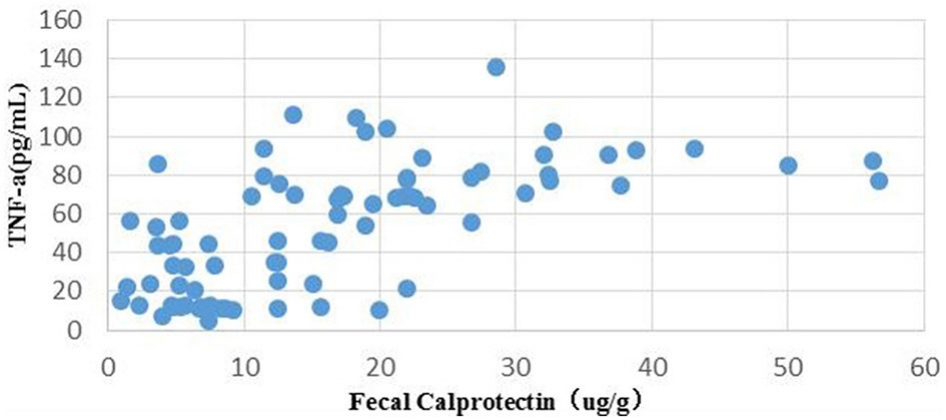
Correlation between serum TNF-α and fecal calprotectin levels in SAE patients. TNF-α, tumor necrosis factor-α; SAE, sepsis-associated encephalopathy.

There was a significant positive correlation between serum TNF-α and fecal calprotectin levels in the SAE group (Figure 2).

### 3.4 Correlation between TNF-α and fecal calprotectin levels and APACHE II and SOFA scores

Serum TNF-α and fecal calprotectin levels of SAE patients were significantly positively correlated with APACHE II (0.287 vs. 0.401) and SOFA scores (0.411 vs. 0.440) (Table 3).

**Table 3:** Correlation between TNF-α and calprotectin levels in serum of SAE patients and APACHE II and SOFA scores.

**Variable**	**APACHE II**	**SOFA**
**TNF-**α		
*r*	0.287	0.401
*P*	0.010	0.001
**Fecal calprotectin**		
*r*	0.411	0.440
*P*	0.001	0.001

### 3.5 Diagnostic value of TNF-α and fecal calprotectin levels for SAE

**Figure 3:**
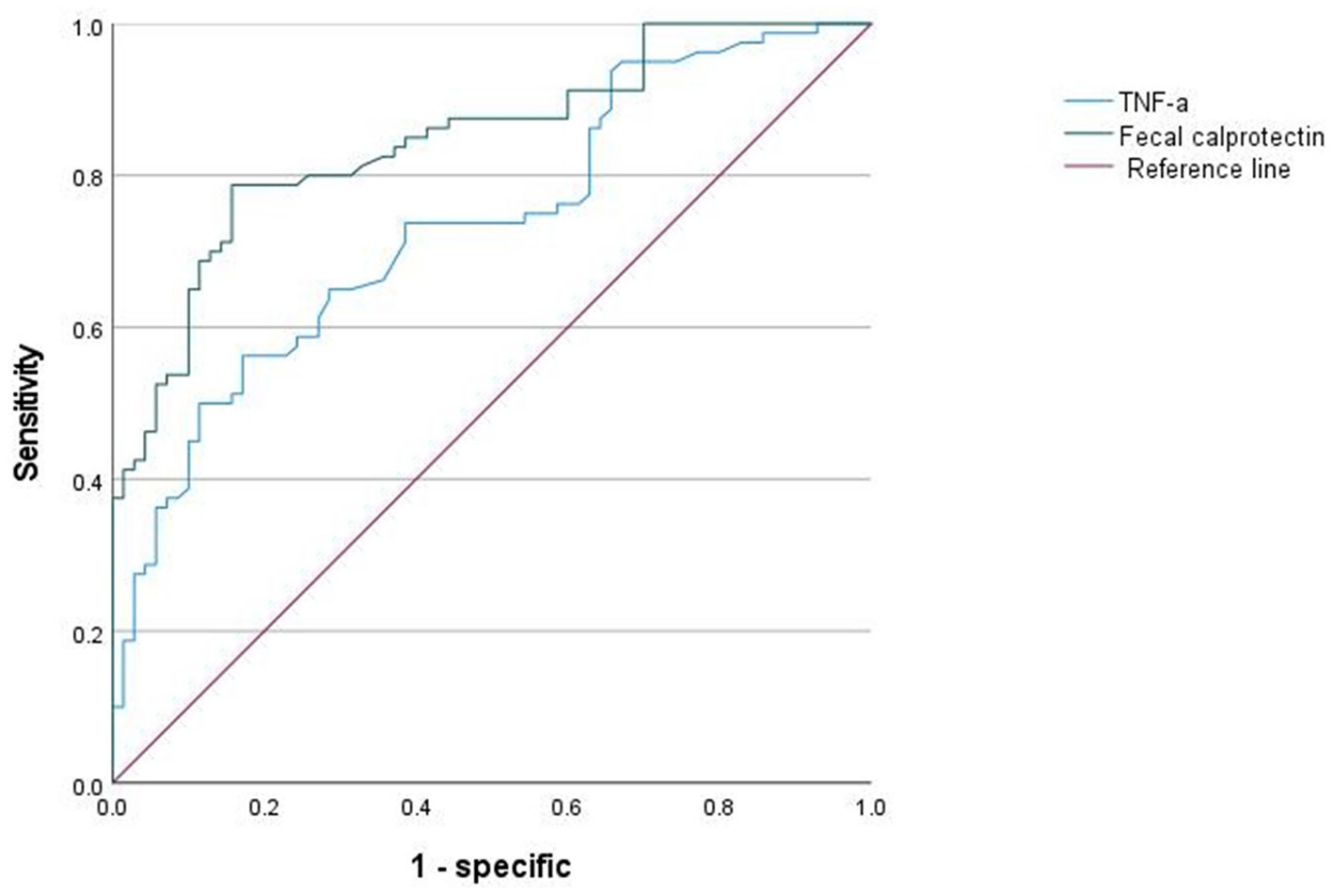
ROC curve of TNF-α and fecal calprotectin in the diagnosis of SAE. ROC, receiver operating characteristic curve; SAE, sepsis-associated encephalopathy.

The predictive value of TNF-α and fecal calprotectin levels was used as the test variable to draw an ROC curve. The area under the curve (AUC) for the diagnostic ability of TNF-α for SAE was 0.735 [95% confidence interval (CI): 0.656–0.814], the cut-off value was 18.95 pg/mL and the specificity and sensitivity were 82.9 and 56.3%, respectively. The AUC of the diagnostic ability of fecal calprotectin level for SAE was 0.847 (95% CI: 0.786–0.908), the cut-off value was 65.25 μg/g and the specificity and sensitivity were 84.3 and 63.1%, respectively. The AUC of TNF-α level combined with fecal calprotectin level for the diagnosis of SAE was 0.914 (95% CI: 0.852–0.976), and both the specificity and sensitivity were 84.3% (Figure 3).

## 4 Discussion

During sepsis, the brain has high oxygen consumption and poor antioxidant and anti-inflammatory capabilities, and the central nervous system is susceptible to damage mediated by oxidative and inflammatory processes. Therefore, up to 70% of patients with sepsis will progress to SAE (14, 15). The pathogenesis of SAE is complex and likely involves multiple factors. Numerous pathological processes occur in parallel and influence each other, each playing a specific role in the development of SAE (16, 17). To date, several pathogenic mechanisms have been identified: oxidative stress, direct nerve damage, and an increase in the level of inflammatory factors (18–20).

During the early stages of septic inflammation, a waterfall-like inflammatory response occurs with the release of a large number of inflammatory mediators. Of the many inflammatory mediators secreted, TNF-α plays a crucial role in the development of severe infection. LPS stimulates monocytes to secrete a large amount of TNF-α, which leads to a significant increase in the concentration of serum TNF-α in sepsis patients. The concentration of TNF-α is associated with the severity of sepsis and prognosis; the higher the TNF-α level, the more critical the condition (6, 21). TNF-α participates in the inflammatory response of SAE. The primary mechanism of TNF-α is to mediate the infiltration of neutrophils in brain tissue and initiate a waterfall-like inflammatory response triggered by a variety of inflammatory factors. TNF-α is highly expressed in the host and plays an important role in the early inflammation stages of sepsis (22). In this study, we found that the TNF-α level of the SAE group was significantly higher than that of the non-SAE sepsis group. Given that TNF-α has a direct causal relationship with the aggravation of brain tissue damage, we speculate that TNF-α mediated the increase in neutrophil infiltration, which led to greater inflammation, the consequent occurrence of SAE and ultimately a high expression of TNF-α.

Calprotectin is a protein derived from neutrophils and monocytes. It is widely distributed in human cells, tissues and bodily fluids, and it can be detected in serum, feces and other specimens (23). Calprotectin has been shown to be involved in the occurrence of SAE. The primary mechanism of calprotectin is the regulation of cytokine secretion via the nuclear factor kappa B pathway and the induction of the transcription of pro-inflammatory factors (e.g., TNF-α, interleukin-6 and interleukin-8), which results in a significant increase in serum TNF-α level and the occurrence of SAE (24, 25). In this study, we showed that the level of fecal calprotectin was significantly higher in the SAE group than in the non-SAE sepsis group, and the calprotectin level was closely related to the occurrence of sepsis or SAE. In addition, the levels of fecal calprotectin and TNF-α were significantly higher in patients in the SAE group than in the non-SAE sepsis group, which was mirrored by the mortality rate. This indicated that both TNF-α and calprotectin are predictive of patient prognosis. We also found high specificity and sensitivity for SAE diagnosis for the combined measure of TNF-α and fecal calprotectin levels.

In this study, we compared the clinical characteristics and outcomes of sepsis patients with and without SAE. We found that patients in the SAE group had significantly higher APACHE II scores, SOFA scores, and 28-day mortality rates compared to the non-SAE group. These findings are consistent with previous research indicating that SAE is associated with worse prognosis in sepsis.

However, a direct comparison of our findings with existing literature reveals that there is a lack of uniformity in the cut-off values used across different studies for severity scoring systems. For instance, studies have reported varying thresholds for APACHE II scores in predicting poor outcomes. Yoon et al. (26) suggested that a cut-off value of 20, the APACHE II score predicted poor neurologic outcomes with a sensitivity of 43.75%, a specificity of 94.12%, a positive predictive value of 94.59%, and a negative predictive value of 41.56%. Similarly, found that SOFA scores ≥15 were strongly correlated with higher ICU mortality (27), especially in patients with multiple organ dysfunction. In our study, although we did not define specific cut-off points, the SAE group consistently had higher median scores, suggesting a more severe disease course.

In terms of predictive performance, the discriminative power of APACHE II and SOFA scores has been widely evaluated using the AUROC. In several studies, the AUROC for APACHE II in predicting mortality in sepsis ranged from 0.70 to 0.85 (28, 29), while SOFA scores often demonstrated AUROC values between 0.80 and 0.91 (30). Though AUROC values were not directly calculated in our study, the significant differences observed between the SAE and non-SAE groups support the established evidence of these scoring systems' prognostic value.

Additionally, some studies have investigated the combined use of scoring systems to enhance predictive accuracy. Combining APACHE II and SOFA scores improved the AUROC to 0.88 (31), outperforming either score used independently. This suggests that future studies should explore integrated scoring models for better stratification of septic patients, particularly those at risk of developing SAE.

Our findings add to the growing body of evidence highlighting the severity of SAE in the context of sepsis. Given the significantly higher mortality in the SAE group, early identification and stratification based on validated scoring systems may be critical in guiding management and improving outcomes.

While it is true that both biomarkers and SAE are associated with more severe forms of sepsis, it is crucial to distinguish between the markers being indicative of sepsis severity in general vs. their specific association with sepsis-associated encephalopathy (SAE). In this study, we observed that patients in the SAE group had significantly higher APACHE II scores, SOFA scores, and mortality rates, which indeed suggest a more severe course of sepsis overall. However, to clarify that the biomarkers and the presence of SAE are more than just indicators of severity, we must consider a few key points:

Specificity of SAE-related biomarkers: while biomarkers like cytokines (e.g., IL-6, TNF-α), S100B protein, and other markers of neuronal injury are elevated in both sepsis and SAE, their levels may be particularly associated with brain dysfunction in sepsis. For example, S100B, a protein that is released from glial cells, has been shown in several studies to be elevated in patients with SAE, particularly when there is neuronal damage or blood-brain barrier disruption (32). These markers are not just general indicators of sepsis severity; they specifically point to neurological involvement, which is a hallmark of SAE. The presence of these biomarkers, in combination with clinical signs of encephalopathy, could therefore indicate that the neurological dysfunction is more than just a byproduct of severe sepsis but a distinct clinical feature of SAE.

The role of cognitive impairment in SAE: unlike general sepsis severity, which is primarily associated with organ dysfunction and overall clinical stability, SAE is characterized by specific neurological manifestations, such as altered mental status, delirium, and cognitive dysfunction. In our study, patients with SAE exhibited a distinct pattern of neurological impairment, which was not merely a reflection of sepsis severity but an indication of brain-specific dysfunction. This cognitive impairment, which was often more pronounced in the SAE group, is an important clinical criterion that helps distinguish SAE from general sepsis.

Comparison with non-SAE sepsis: the key difference between the SAE and non-SAE groups in our study lies not only in the severity of sepsis but also in the distinct neuroinflammatory response and neuronal injury markers. While patients in both groups had high levels of markers of systemic inflammation, the SAE group exhibited additional evidence of brain involvement, such as elevated levels of neurodegeneration-related biomarkers (e.g., neurofilament light chain or tau proteins) that are specifically associated with neuronal injury rather than just systemic inflammation. This suggests that, beyond the general severity of sepsis, SAE-specific mechanisms such as neuroinflammation and blood-brain barrier dysfunction are at play, further confirming that the biomarkers indicate SAE rather than simply the severity of sepsis.

Biomarker-driven diagnosis of SAE: several studies have suggested that biomarkers related to neuroinflammation (such as IL-1β, IL-6, and TNF-α) and neuronal injury (like S100B, GFAP, and neurofilament proteins) are more specifically elevated in patients with SAE than in those with severe sepsis without encephalopathy. The elevated levels of these biomarkers in our study align with these findings, providing further evidence that the biomarkers are not merely reflecting the severity of sepsis as a whole but are specifically linked to the pathophysiology of SAE.

Clinical implications: the differentiation between sepsis severity and SAE-specific biomarkers has important clinical implications. If biomarkers such as S100B or GFAP are elevated, clinicians can use these markers not only as indicators of severe sepsis but also as potential flags for brain involvement. This can help guide therapeutic strategies aimed at mitigating neuroinflammation and protecting neuronal function in patients with SAE, an aspect that would not be addressed if biomarkers were solely viewed as general indicators of sepsis severity.

## 5 Conclusions

In summary, TNF-α and fecal calprotectin are closely related to the occurrence of SAE and may be involved in the pathogenesis of SAE. TNF-α level combined with fecal calprotectin level has high specificity and sensitivity for the diagnosis of early SAE. Moreover, they have good predictive value and may serve as an indicator of patient prognosis. However, our sample was small, and thus, further research using a larger sample size is needed.

## 6 Limitations

Despite the valuable findings in this study, several limitations should be acknowledged. First, the study was conducted at a single center, which may limit the generalizability of the results to other healthcare settings or populations. A multi-center study with a larger and more diverse sample would help to validate the findings and enhance the external validity. Second, the study design is observational and cross-sectional, which makes it difficult to establish causality between the biomarkers (TNF-α and fecal calprotectin) and the development or progression of sepsis-associated encephalopathy (SAE). Longitudinal studies are needed to better understand the temporal relationship between these biomarkers and SAE outcomes. Additionally, while TNF-α and fecal calprotectin were found to correlate with disease severity, other potential confounding factors, such as comorbidities, medication use, and individual patient characteristics, were not fully controlled for in the analysis. This could have influenced the results and may require further investigation in future studies. Moreover, the study focused primarily on the early phases of sepsis, and thus, the biomarkers' predictive value for long-term outcomes remains unclear. Finally, although TNF-α and fecal calprotectin showed promising results, their role in clinical practice would need to be further confirmed through prospective trials with larger sample sizes to assess their potential as routine diagnostic and prognostic tools.

## Data Availability

The raw data supporting the conclusions of this article will be made available by the authors, without undue reservation.
